# Land-Cover Changes to Surface-Water Buffers in the Midwestern USA: 25 Years of Landsat Data Analyses (1993–2017)

**DOI:** 10.3390/rs12050754

**Published:** 2020-02-25

**Authors:** Tedros M. Berhane, Charles R. Lane, Samson G. Mengistu, Jay Christensen, Heather E. Golden, Shi Qiu, Zhe Zhu, Qiusheng Wu

**Affiliations:** 1 Pegasus Technical Services, Inc., c/o U.S. Environmental Protection Agency, Cincinnati, OH 45219, USA; 2 Office of Research and Development, U.S. Environmental Protection Agency, Cincinnati, OH 45268, USA; 3 National Research Council, c/o U.S. Environmental Protection Agency, Cincinnati, OH 45268, USA; 4 Department of Natural Resources and the Environment, University of Connecticut, Storrs, CT 06269, USA; 5 Department of Geography, University of Tennessee, Knoxville, TN 37996, USA

**Keywords:** Continuous Change Detection and Classification (CCDC), development, Landsat, land use/land cover (LULC) change, p23r31, p23r32, p24r33, random forest, size class, surface-water buffers, time series, wetland

## Abstract

To understand the timing, extent, and magnitude of land use/land cover (LULC) change in buffer areas surrounding Midwestern US waters, we analyzed the full imagery archive (1982–2017) of three Landsat footprints covering ~100,000 km^2^. The study area included urbanizing Chicago, Illinois and St. Louis, Missouri regions and agriculturally dominated landscapes (i.e., Peoria, Illinois). The Continuous Change Detection and Classification algorithm identified 1993–2017 LULC change across three Landsat footprints and in 90 m buffers for ~110,000 surface waters; waters were also size-binned into five groups for buffer LULC change analyses. Importantly, buffer-area LULC change magnitude was frequently much greater than footprint-level change. Surface-water extent in buffers increased by 14–35x the footprint rate and forest decreased by 2–9x. Development in buffering areas increased by 2–4x the footprint-rate in Chicago and Peoria area footprints but was similar to the change rate in the St. Louis area footprint. The LULC buffer-area change varied in waterbody size, with the greatest change typically occurring in the smallest waters (e.g., <0.1 ha). These novel analyses suggest that surface-water buffer LULC change is occurring more rapidly than footprint-level change, likely modifying the hydrology, water quality, and biotic integrity of existing water resources, as well as potentially affecting down-gradient, watershed-scale storages and flows of water, solutes, and particulate matter.

## 1. Introduction

Land use and land cover (LULC) progression in the Midwestern United States (US) has changed from historically widespread tallgrass prairie and forested landscapes to intensively managed agricultural lands and highly developed urban landscapes [1-5]. This massive transformation has made the Midwestern US one of the most agriculturally productive regions of the US, where nine states (Illinois, Indiana, Iowa, Kansas, Minnesota, Missouri, Nebraska, Ohio, and South Dakota) are responsible for 76% of the total US crop production [6]. Similarly, urbanization has altered the landscape; between 1940 and 2000 the number of housing units in the Midwestern US grew by 146%, with much of the urbanization occurring in areas peripheral to the metropolitan centers [7].

However, LULC change such as that experienced in the Midwestern US also dramatically alters local- to continental-scale landscape structure, function, connectivity, productivity, and processes [8]. Specific to aquatic resources, research has demonstrated that LULC change affects hydrological functions, including stream discharge [9], peak flows [10], surface runoff [11], water levels [12], and sedimentation rates [13-15]. The impacts of LULC change at local to regional scales can further affect watershed-scale biogeochemical processing rates, with concomitant impacts on water quality [16]. Loss of “bioreactive” landscape elements [17], such as wetlands and similar waters, and transformation to different LULC types may cumulatively increase nutrient loading to down-gradient systems [4,18-22].

The effects of anthropogenic alterations on down-gradient biogeochemical concentrations can vary based on waterbody size classes. For instance, Evenson et al. [22] reported that the removal of smaller water bodies (e.g., drained or filled) across a watershed could substantially alter watershed-scale water residence times, which is also likely to affect biogeochemical processing rates. In a literature review of over 600 papers, Cheng and Basu [23] determined that smaller water bodies are more biogeochemically reactive than larger systems. Van Meter and Basu [24], Serran and Creed [25], and Creed et al. [19] have reported that small aquatic systems, such as wetlands, are more likely to experience LULC change (i.e., be hydrologically altered or destroyed), thereby disproportionately affecting watershed-scale hydrological characteristics and biogeochemical processing rates.

Modifications to the areas buffering surface waters can also have profound impacts on the water quantity and quality in a given water body [13,14,26-28]. Buffers affect water quality and quantity through energy dissipation and the physical settling of entrained particles. Furthermore, settled particles and solutes can undergo biogeochemical processing in areas buffering water bodies [29,30]. Hence, changes to the buffers (e.g., width, structure) can harbinger state changes affecting aquatic resources [26,31,32], as well as affecting the condition of downstream systems [8].

Remotely sensed LULC change data over time provide informative LULC analyses and syntheses useful for managing watersheds and the water bodies of all sizes that remain therein. However, these analyses tend to focus on gross image-wide changes rather than on the effects of LULC change in areas buffering surface-water bodies [26]. Thus, the signal of LULC change in buffering areas may be lost in the “noise” of LULC change occurring elsewhere across the broader landscape. This signal dampening affects both how we quantify the functions and ecosystem services provided by areas buffering the remaining surface water resources (e.g., [8]) and how shifting LULC changes affect these waters themselves (e.g., [26]). Research advances are therefore needed to understand how—and to what extent—surface water buffers are modified in the broader landscape mosaic of LULC changes.

To address these research gaps, we conducted an analysis of temporally continuous LULC change across a 25 year time period in the Midwestern US. The analysis covers three Landsat footprints including the developing urban lands of Chicago, Illinois, and St. Louis, Missouri, as well as the predominantly agricultural areas surrounding the smaller urban center of Peoria, Illinois. We explored not only 25 consecutive years of footprint-wide change, but also analyzed changes to buffers surrounding different surface-water body size classes. Specifically, we sought to answer the following research questions:

1. What is the magnitude, spatial distribution, and annual rate of LULC change across three Landsat footprints (nearly 100,000 km^2^) of the Midwestern US?
2. How has LULC change occurred over time in buffers surrounding different water body sizes within the three study areas?

We hypothesized that urbanizing trends would continue apace, and hence LULC change would be greatest in the urban-dominated Landsat footprints (i.e., Chicago, Illinois, and St. Louis, Missouri; [33]). As smaller water bodies are more frequently altered as a result of landscape drainage and filling [13,19], we further expected that the smallest waters in our study, those < 0.1 ha in area, would experience the greatest LULC change in their peripheral buffers. We conducted our analysis using the Continuous Change Detection and Classification algorithm (CCDC, [34,35]) and ~1750 Landsat images for our three study areas, incorporating 35 years of data.

## 2. Materials and Methods

### 2.1. Study Area

**Figure 1.**
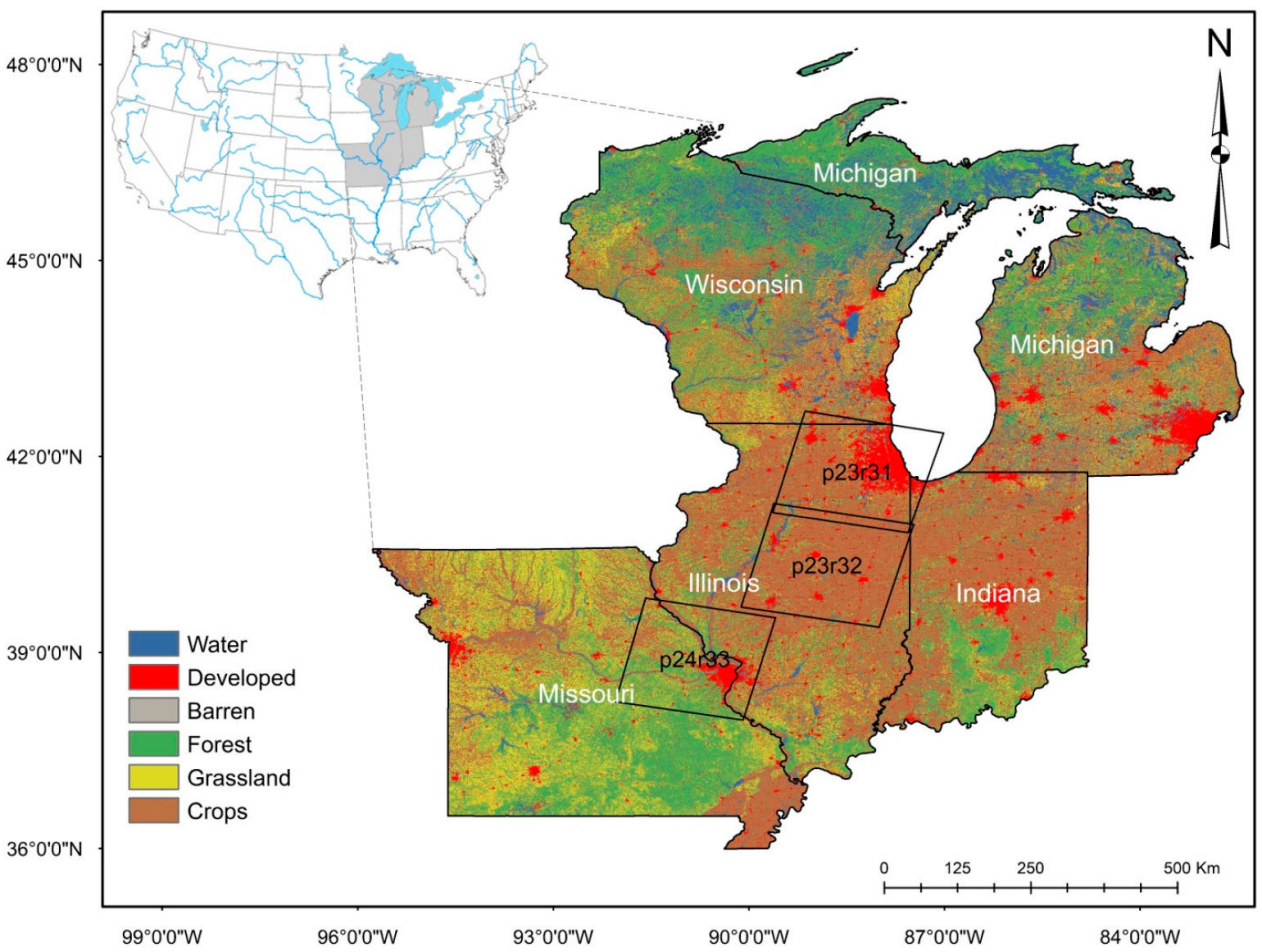
Midwestern US study area encompasses over 100,000 km^2^, including urbanizing Chicago, Illinois (p23r31) and St. Louis, Missouri (p24r33) regions, as well as rural areas of central Illinois (including Peoria, the developed area in the northwestern corner of p23r32). The background land use/land cover (LULC) data are from the 2001 national land cover dataset (NLCD) [41], binned into gross LULC classes, as described in Section 2.3.2, below.

The Midwestern US comprises ~25% of the conterminous US by land area and contains 22% of the nation’s population [36]. The regional climate consists of cold and long winters, with wet springs and temperate summers [37]. However, global climate change models predict warmer and wetter fall-through-spring seasons, with notably drier summer months [38]. The study area is almost entirely in the Major Land Resource Area “Central Feed (Grains and Livestock Region” [37] and has level to gently sloping landscapes, much of which consist of the formerly glaciated plains. Our Midwestern study area (Figure 1) includes three Landsat footprints (World Reference System path (p) and row (r) of p23r31, p23r32, and p24r33). The area represented by the three Landsat footprints has both major agricultural production zones and dense population centers [6,7]. As a result, approximately 85% of the pre-European settlement wetlands and similar waters have been lost from the region to agricultural drainage and urban development [39,40]. The urban centers include the cities of Chicago (Illinois) and St. Louis (Missouri) in p23r31 and p24r33, respectively, as well as smaller urban centers in p23r32 (e.g., the Illinois cities of Peoria, Bloomington, Champaign, Decatur, and Springfield). For simplicity, we will hereafter refer to the footprints, from north to south, as Chicago for p23r31, Peoria for p23r32, and St. Louis for p24r33.

### 2.2. Data Acquisition and Preprocessing

#### 2.2.1. Landsat Data Downloading and Preprocessing

**Figure 2.**
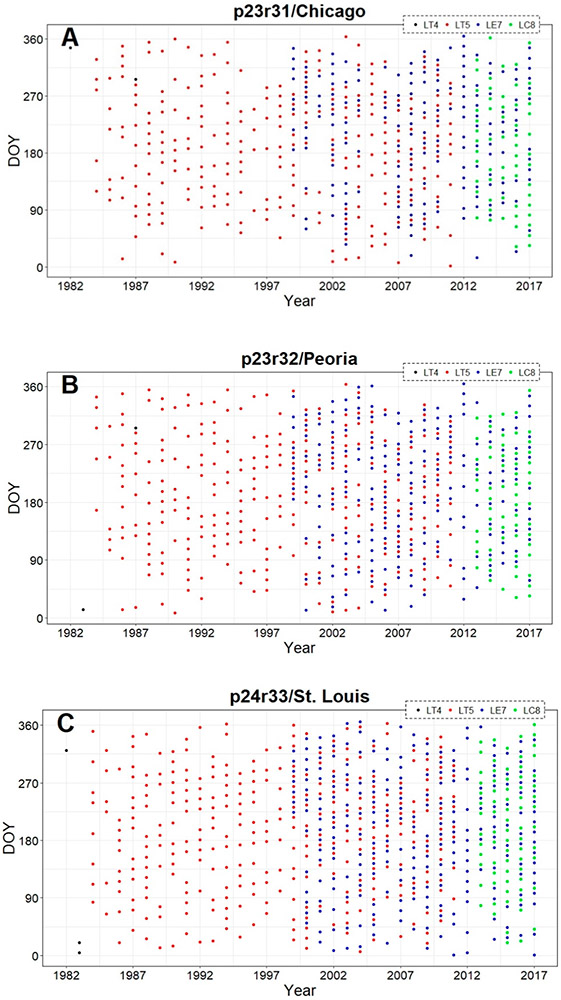
Distribution of Landsat data utilized for LULC change by footprint, sensor type, year and Julian date of the year (DOY) for (**A**) Chicago (p23r31), (**B**) Peoria (p23r32), and (**C**) St. Louis (p24r33).

We downloaded geometrically and atmospherically corrected (i.e., USGS “Level 1T”) Landsat 4 and 5 Thematic Mapper (TM), Landsat 7 Enhanced Thematic Mapper Plus (ETM+), and Landsat 8 Operational Land Imager (OLI) data, including all imagery with ≤ 80% cloud-coverage by scene area from the United States Geological Survey-Earth Resources Observation and Science online portal [42,43]. We downloaded a total of 1749 images for the Chicago, Peoria, and St. Louis footprints covering the period 1982-2017, distributed evenly across the years and seasons (Figure 2A-C). We converted raw digital numbers to surface reflectance (bands 1–5, 7) and brightness/surface temperature (band 6) values using the Landsat Ecosystem Disturbance Adaptive Processing System algorithm [44]. We used the GERSLab [45] preprocessing tool to stack the six spectral bands, one thermal band, and one Landsat Level-1 pixel Quality Assessment (QA) or Landsat C version of Function of Mask band (CFMask, providing information about the quality of the pixels in terms of clouds, cloud shadows and snow contaminations [46-48]), for the analyses described below.

#### 2.2.2. Land Cover Data Downloading and Processing

We acquired thematic land cover classification maps from the National Land Cover Dataset (NLCD) for 2001, 2006 and 2011 from the USGS portal [43]. We used the 2001 NLCD in the land cover classification, described in Section 2.3, and compared our classification with the three NLCD products, described in Section 2.3.3. The NLCD maps were cropped to the Landsat footprint boundary and re-projected to a common coordinate system (WGS 1984, UTM Zones 15N for St. Louis and 16N for Chicago and Peoria).

### 2.3. Change Detection Model Implementation and Assessment

#### 2.3.1. Continuous Change Detection and Classification (CCDC) algorithm

We analyzed regional time-series LULC change analysis using CCDC, which uses the Robust Iteratively Reweighting Least Squares (RIRLS) method to iteratively fit observed data to the LULC dynamic time-series model that incorporates seasonality, trend (for gradual changes), and breaks (for abrupt changes) [34]. We used CCDC version 13.01 (64-bit Linux-machine version) downloaded from Global Environmental Remote Sensing (GERS) Laboratory [45]. The model identifies breaks or transitions using a data-driven thresholding approach on the included Landsat bands [34]. Breaks or changes are identified in CCDC when the spectral signature deviates from the fitted model predictions in consecutive dates of images [34,49]. Further CCDC application fundamentals and details of its implementation can be found in Zhu and Woodcock [34].

#### 2.3.2. Random Forest Training and Classification

We classified the CCDC model output data with version 1.02 of the CCDC Assistor tool [45] within the CCDC environment using random forest [50]. Inputs to the supervised classification included time-series model coefficients and the root mean square error (RMSE) values used to generate a continuous land cover map of the modeling domain [34]. The 2001 NLCD dataset was used to train the classifier using 20,000 pixels distributed proportionally across the original 15 land-cover classes based on the optimized training data selection strategy [47]. Accordingly, the approach uses 600–8000 pixels per class [47,51].

We subsequently binned the continuous land-cover classification maps produced from the CCDC and the NLCD data into six major land cover types for our analyses (see Figure 1): **water** (NLCD classes of open water, woody wetlands, emergent herbaceous wetlands), **forest** (NLCD classes of deciduous forest, evergreen forest, mixed forest, and shrub/scrub), **grassland** (NLCD classes of grassland/herbaceous, pasture/hay), **crops** (NLCD class of cultivated crops), **developed** (NLCD classes of developed—open space, developed—low intensity, developed—medium intensity, developed—high intensity), and **barren** (NLCD class of barren land—rock/sand/clay).

#### 2.3.3. Classification Agreement with NLCD

We evaluated the spatiotemporal performance of the CCDC algorithm and subsequent random forest classification over time through the generation of both omission and commission error metrics.

We assessed classification correspondence with the 2001, 2006, and 2011 NLCD datasets [52-54]. We kept a minimum linear separation distance between any two points of 250 m (a distance selected to decrease autocorrelation potential) that resulted in nearly 238,000 randomly distributed points across the three footprints. We assessed classification agreement by (1) extracting the CCDC-derived class for each pixel using July 1st of each year (thereby avoiding untilled agricultural lands and snow-covered landscapes; [51]), and (2) developing confusion matrices contrasting the CCDC class with 2001, 2006, and 2011 NLCD thematic data that we binned into major land-cover types (described above in 2.3.2).

### 2.4. Data Analyses

We analyzed the annual LULC change for a 25-year period from 1993–2017 at both the image-scale and within a 90 m buffering distance around surface waters, a distance that approximates a commonly used 100 m buffer for aquatic monitoring and assessments [31,55] and one that seamlessly comports to the 30 m Landsat pixel size. The dataset from the period 1982–1985 was used for model initiation and a six year CCDC output window (1986–1991) was used to identify “waters” from the random forest classification (i.e., including open water, woody wetlands, and emergent herbaceous wetlands, as described above). A conservative rubric was applied, such that a pixel was required to be classified as water in at least five of six years between the period 1986–1991 to be considered “water”; this decreased the likelihood that climatic vagaries and/or mixed-pixel (“edge effects”) would result in a pixel being misidentified as water. We used 1992 imagery for final model initiation and conducted LULC change analyses from the period 1993–2017. Waters ranged in size from a single pixel (0.09 ha) to 58,850 ha in size across the three images. We excluded Lake Michigan, a 4000 km^2^ water body (the portion within our Chicago footprint), from all analyses of the Chicago footprint.

We calculated the LULC change over 25 years using three approaches: (1) footprint-level time-series LULC change, (2) water body buffer time-series LULC change within 90 m buffers for each footprint, and (3) water body size-class analyses of time-series LULC change within a 90 m water body buffers (i.e., a post-hoc approach analyzing each water body uniquely). We conducted the footprint-scale 90 m assessment by first buffering each water body (as determined above) within a GIS environment. We then identified the corresponding 90 m buffer LULC pixels from the CCDC output for each year using R (using R packages raster, rgdal, rgeos, and sf [56-59]). For the third analysis, each water body had a unique 90 m buffer, with LULC calculated and exported for analyses. For the last analysis, we calculated the area of each water body within a GIS environment and subsequently analyzed LULC change over time, following Christensen et al. [40], binning surface water features into five size-based categories: (a) < 0.1 ha, (b) 0.1 to < 1.0 ha, (c) 1.0 to < 10.0 ha, (d) 10.0 to < 100.0 ha, and (e) ≥ 100 ha.

## 3. Results

### 3.1. Thematic Classification Characterization

Confusion matrices (Tables 1-3) calculated an overall agreement of 81%–89% across all three footprints and three NLCD datasets (2001, 2006, and 2011). Peoria (footprint p23r32) had the highest overall correspondence of ~89%, whereas Chicago (footprint p23r31) and St. Louis (footprint p24r33) had overall agreements of 84% and 81%, respectively. In general, water, developed, forest, and cropland cover classes were more accurately classified, and grassland and barren classes were more poorly classified. Misclassification errors with grassland and barren classes likely reflect the thresholding process inherent within the NLCD (e.g., barren require <15% vegetation, whereas grasslands require >80% vegetation) and approximate reported regional misclassification errors within the NLCD [41,60]. The small differences in overall agreement between years suggest that the model performed well over time (e.g., overall agreement in Chicago footprint for the three time periods was 84.1% in 2001, 84.0% in 2006, and 83.8% in 2011).

### 3.2. Spatiotemporal LULC Change Dynamics at Footprint Level

**Figure 3.**
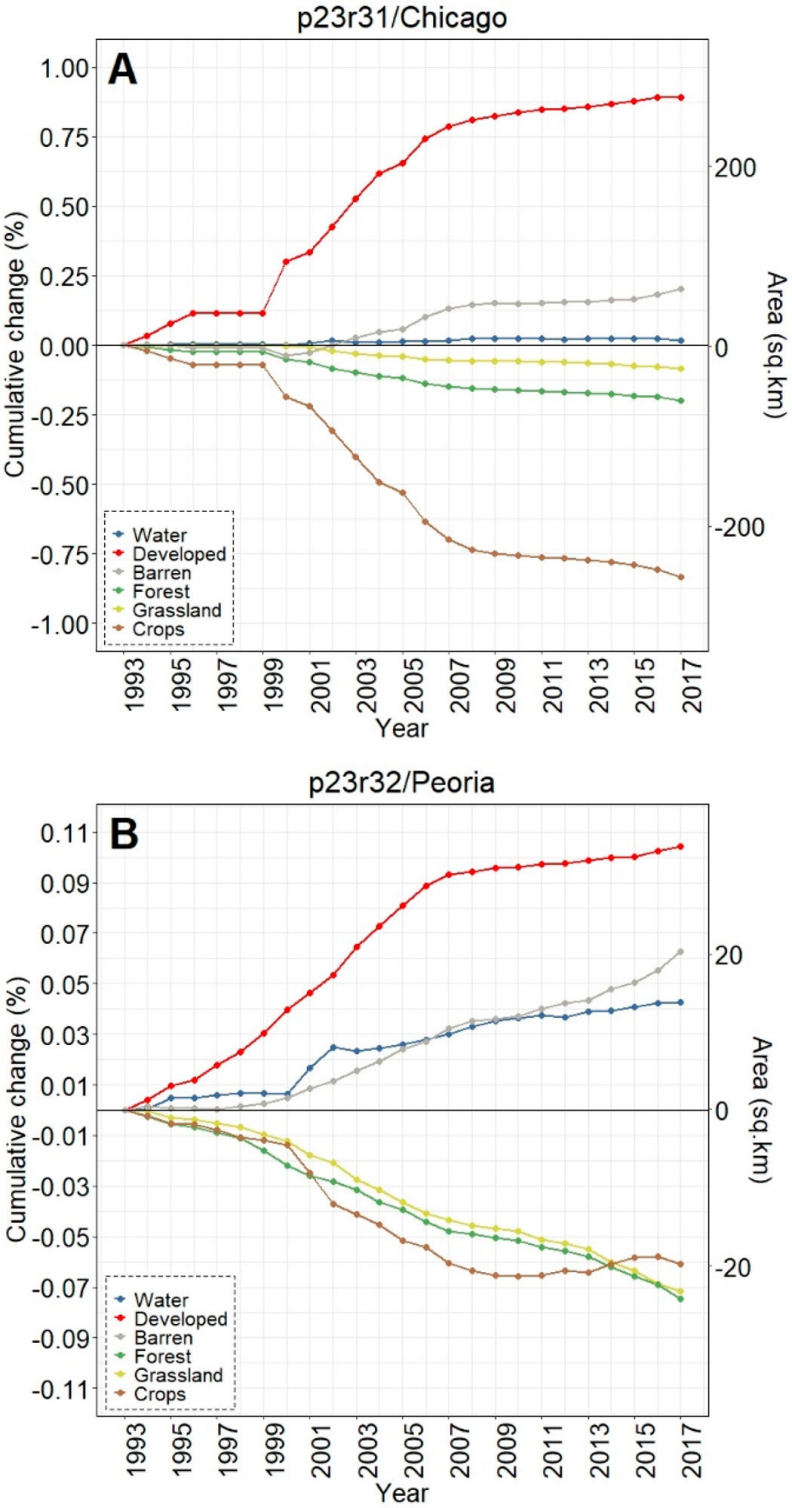

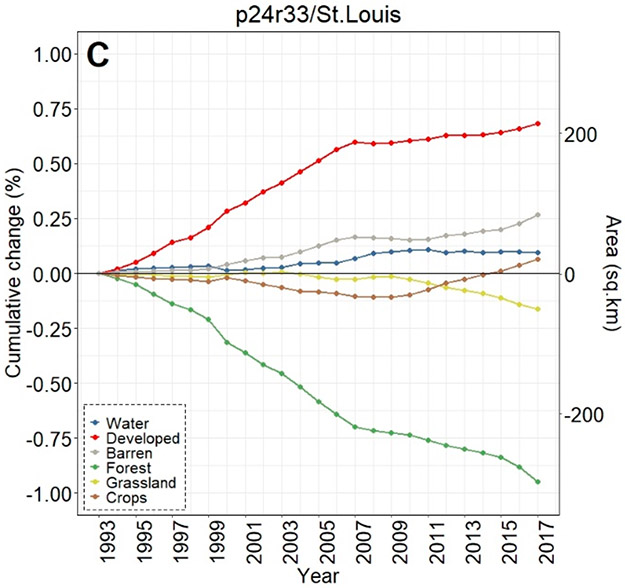
Cumulative footprint-level LULC change, both in percentage and approximate area, between the period 1993–2017 for (**A**) Chicago, (**B**) Peoria, and (**C**) St. Louis footprints. Our areal analyses throughout were calculated on pixel-change; we did not use a reference classification [61]. Note the differences in y-axis values across panels A–C.

Across all three footprints, developed lands cumulatively increased over 25 years of analyses, while there were concomitant decreases in croplands in the Chicago footprint (Figure 3A), croplands/grasslands/forested lands in the Peoria footprint (Figure 3B), and forested land cover in the St. Louis footprint (Figure 3C). Whilst the percentages of LULC change may be relatively small (e.g., ~1% in 25 years), the actual areal change is substantive—at least for the Chicago and St. Louis footprints. For example, there was a 25 y increase of 280 km^2^ in developed lands in the Chicago footprint (Figure 3A), while nearly 300 km^2^ of forested lands in the St. Louis area were converted to other land cover types (see Figure 3C). Developed lands in the Peoria footprint increased ~36 km^2^; all other changes at the footprint level in the Peoria analyses were <30 km^2^ (or <0.10%). All three footprints showed increases in water and barren lands. Interestingly, the St. Louis footprint crop LULC change decreased through ~2009 before demonstrating an increased cropland trend around 2009, ultimately resulting in a slight positive increase in croplands (see Figure 3C).

**Figure 4.**
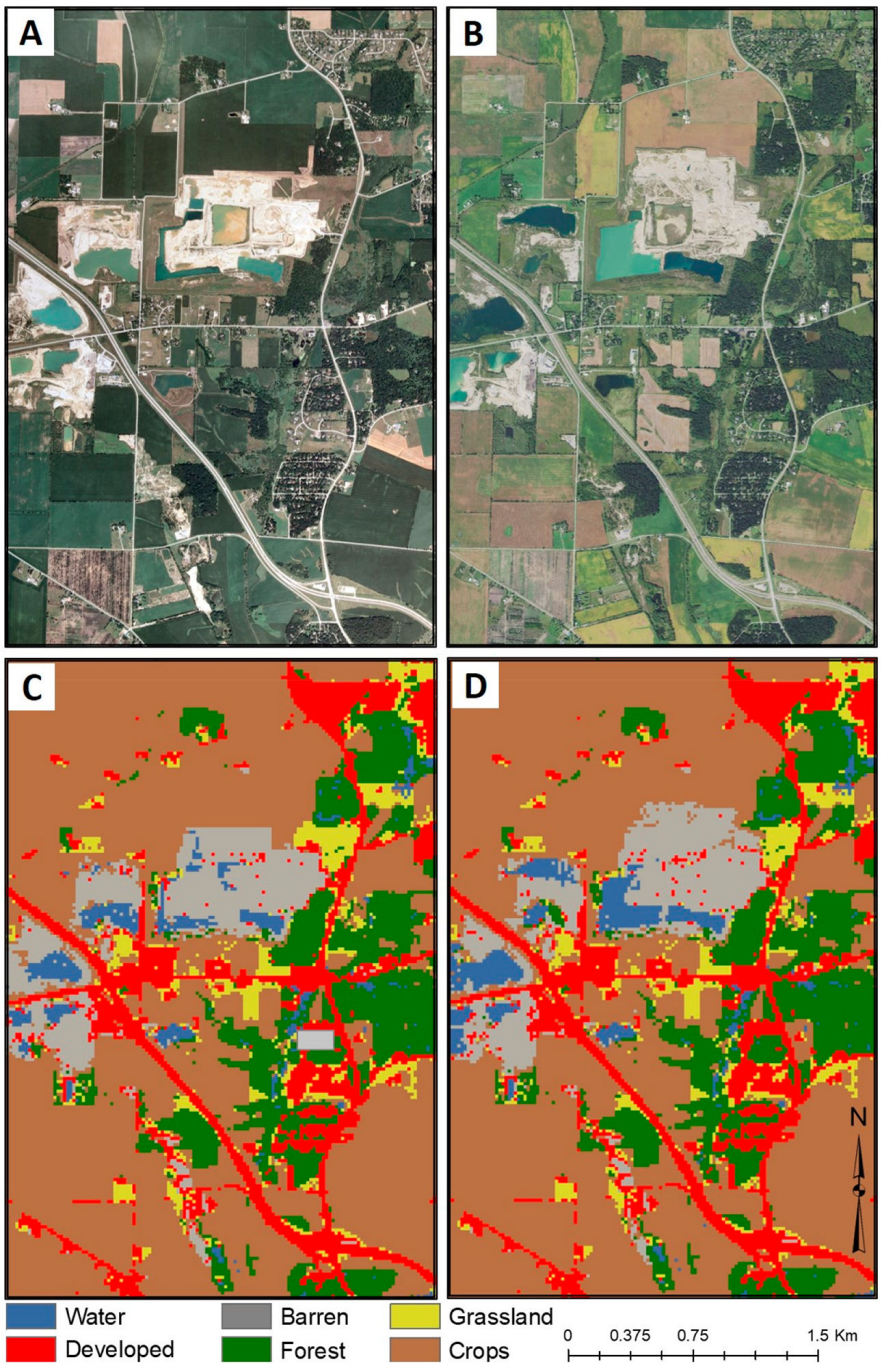
Example of Chicago footprint LULC change between two points in time, (**A**) National Agricultural Inventory Program (NAIP, 1 m resolution) image from 21 July 2007, (**B**) NAIP image from 21 September 2015, (**C**) CCDC map contemporaneous with (A), from 1 July 2007, and (**D**) CCDC map contemporaneous with (B), from 1 July 2015.

**Figure 5.**
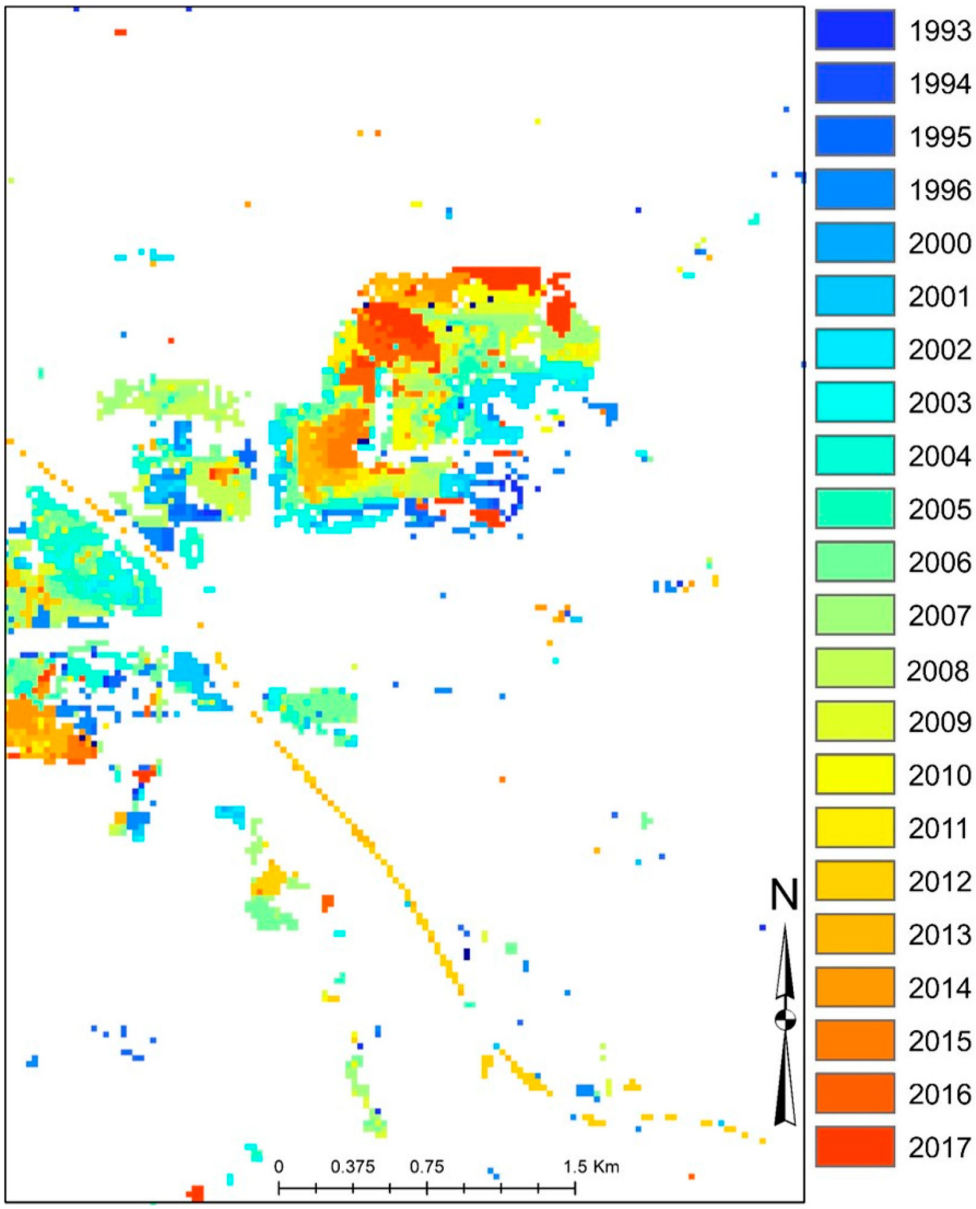
CCDC-informed year-of-LULC change in Figure 4; the expansive barren area in the center of the image appears to be a quarry that substantially expanded in the period from ~2008 onwards.

Dynamic change over time is evident in Figure 3A-C using CCDC; Figure 4A-D shows an LULC change in a representative area between two points (2007 and 2015). Figure 5 provides information on the spatiotemporal “year of change”, demonstrating the power of time-series analyses to identify the temporal nature of LULC change.

### 3.3. Spatiotemporal LULC in Surface Water Buffers

#### 3.3.1. LULC Change in Water Body Buffers Compared to Footprint-Scale

**Figure 6.**
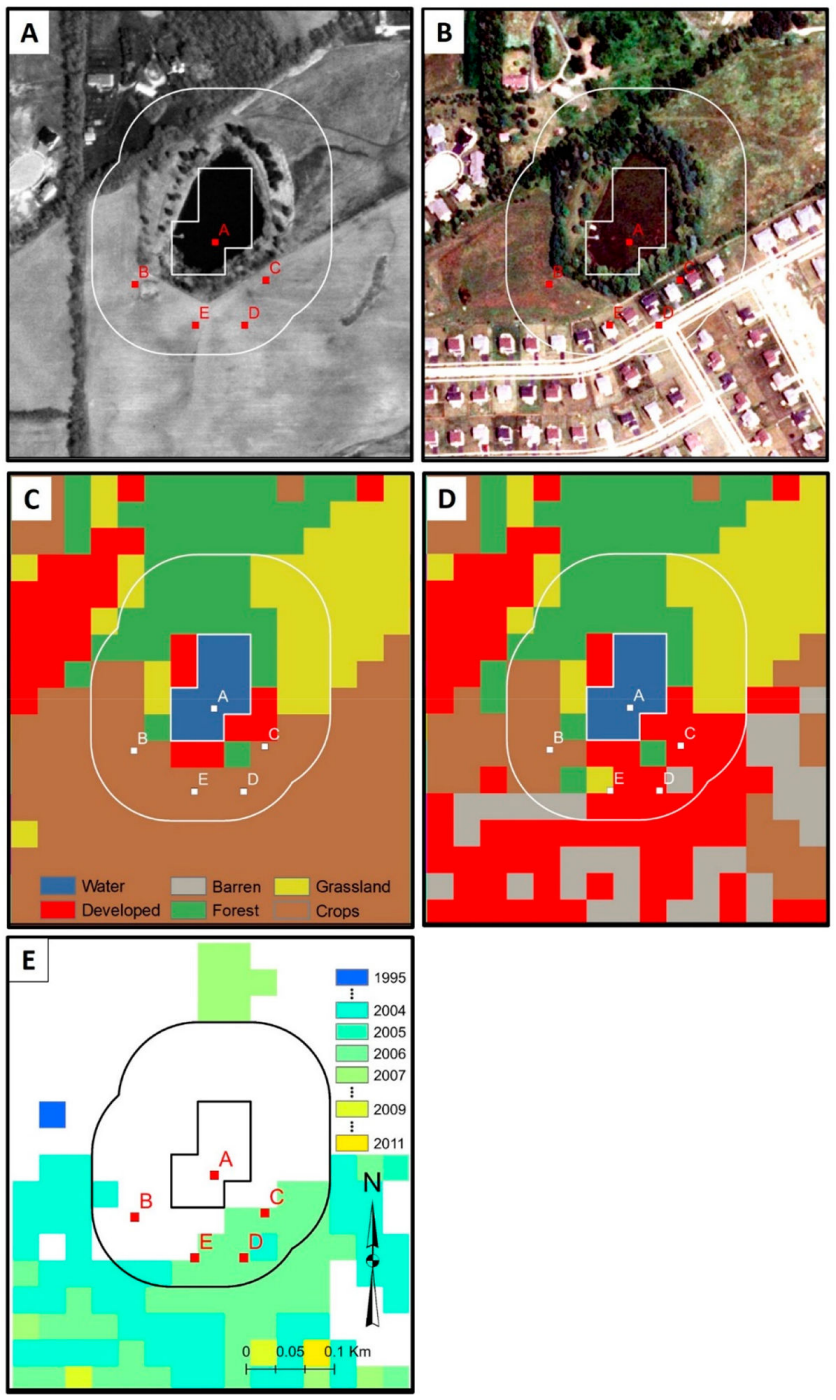
LULC change within a 90 m water body buffer, (**A** aerial photo of surfaces water in Yorkville, IL dated 05 April 1998, (**B**) aerial photo from 06 August 2009 after significant development, (**C**) CCDC LULC classification of July 01, 1998, (**D**) CCDC LULC classification of 01 July 2009, (**E**) CCDC-informed year of change (see Figure 7 for further information on inset points A–E).

**Figure 7.**
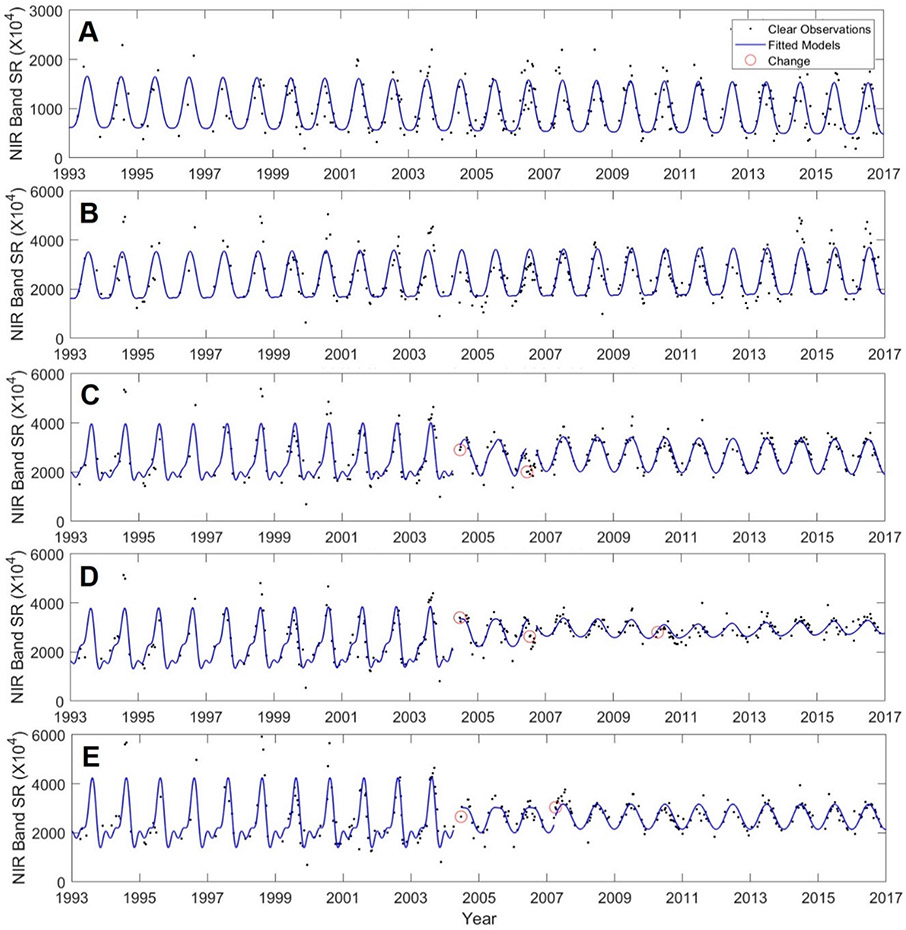
LULC change occurred to the select pixels identified in Figure 6 ~2004, as indicated by the break in the CCDC-fitted RIRLS models, (**A**) water body (no change indicated), (B) crops (no change indicated), (**C**) LULC change (from crop) to developed class), (**D**) LULC change (from crop to developed class), (**E**) LULC change (from crops to grassland). The panels in Figure 7 align with the five points (A–E) given in Figure 6.

We identified 51,619 surface waters in Chicago, 22,186 in Peoria, and 34,475 in St. Louis footprints, covering 3.9%, 2.1%, and 3.3% of each footprint, respectively. Adding the 90 m buffer resulted in the buffer analyses covering approximately 8.2% of Chicago, 4.2% of Peoria, and 6.3% of the St. Louis footprint areas. LULC change over the 25 year horizon occurred in the buffers of 41% of waters in the Chicago-area footprint, 30% in the Peoria-area footprint, and 51% in the St. Louis-area footprint. In Figure 6A-E, LULC change within the buffer surrounding a water body in the Chicago-area footprint is evident, with development encroaching upon the water body (e.g., Figure 6E). Change to the sinusoidal CCDC curves for select points within Figure 6 are presented in Figure 7A-E; indications of LULC change-points across the 25 year time-period for pixels within the areas buffering the water body have evidently occurred ~2004.

**Figure 8.**
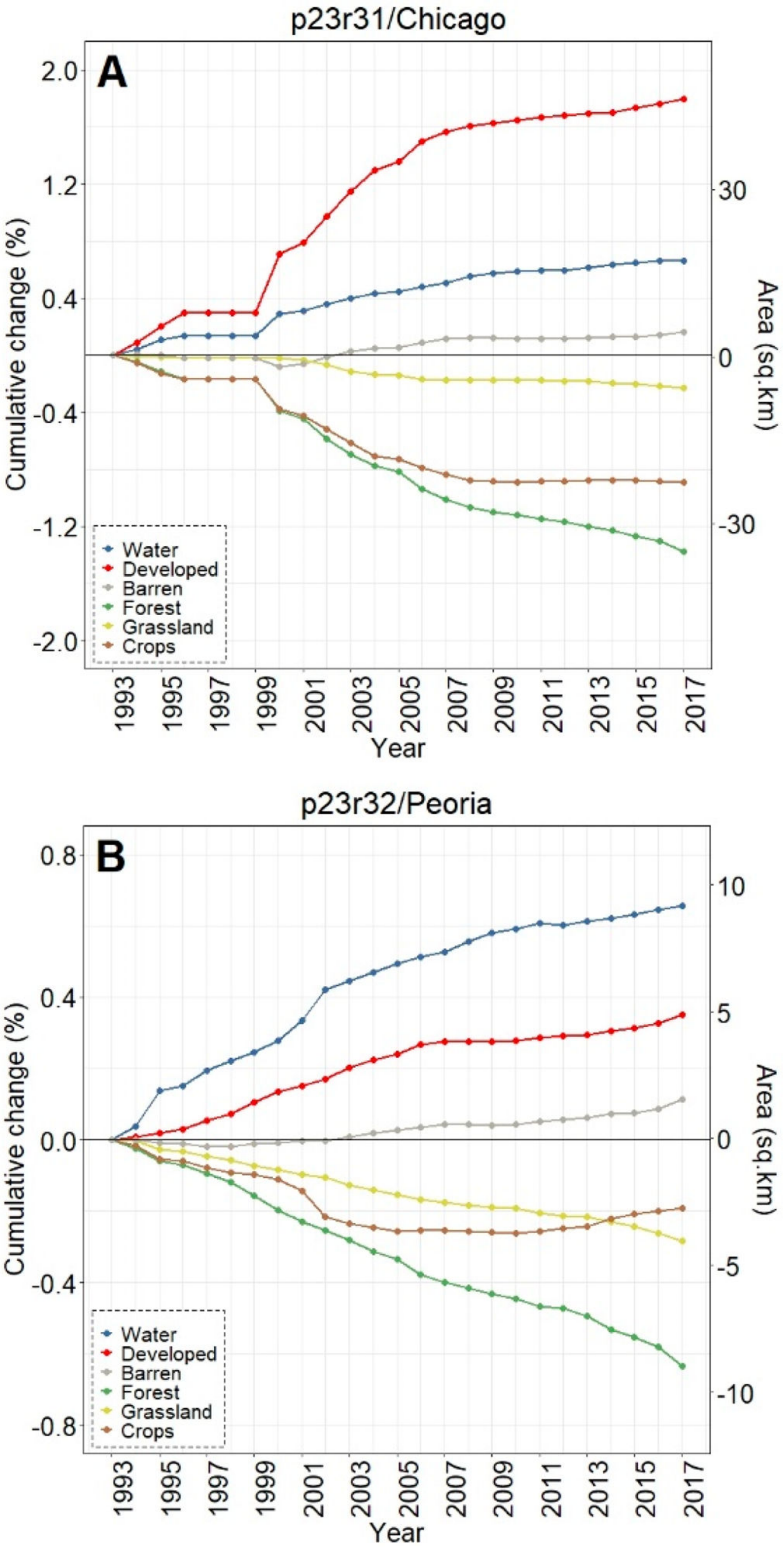

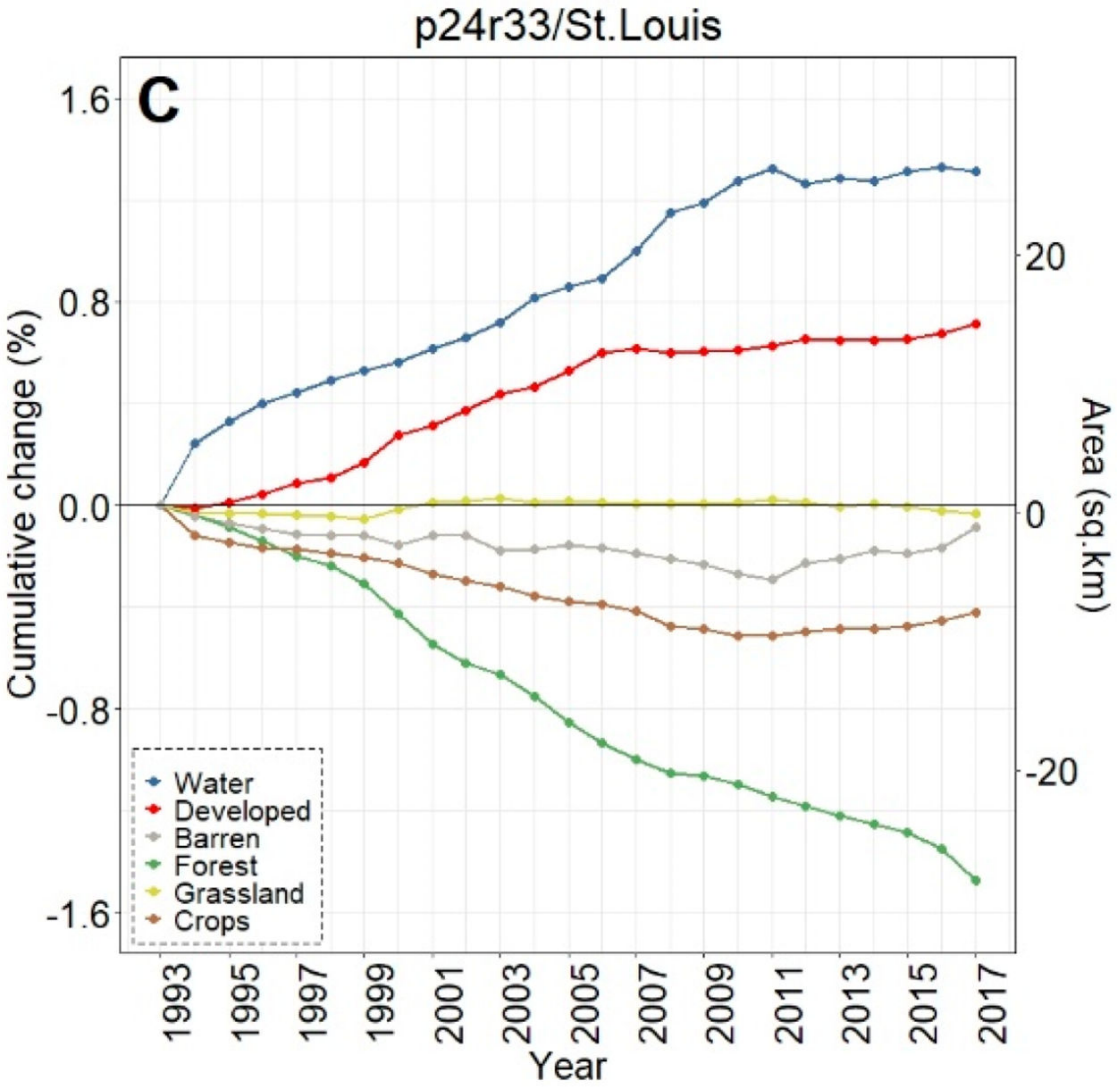
Cumulative LULC change in 90 m areas buffering surface waters between the period 1993–2017 for (**A**) Chicago, (**B**) Peoria, and (**C**) St. Louis footprints.

**Figure 9.**
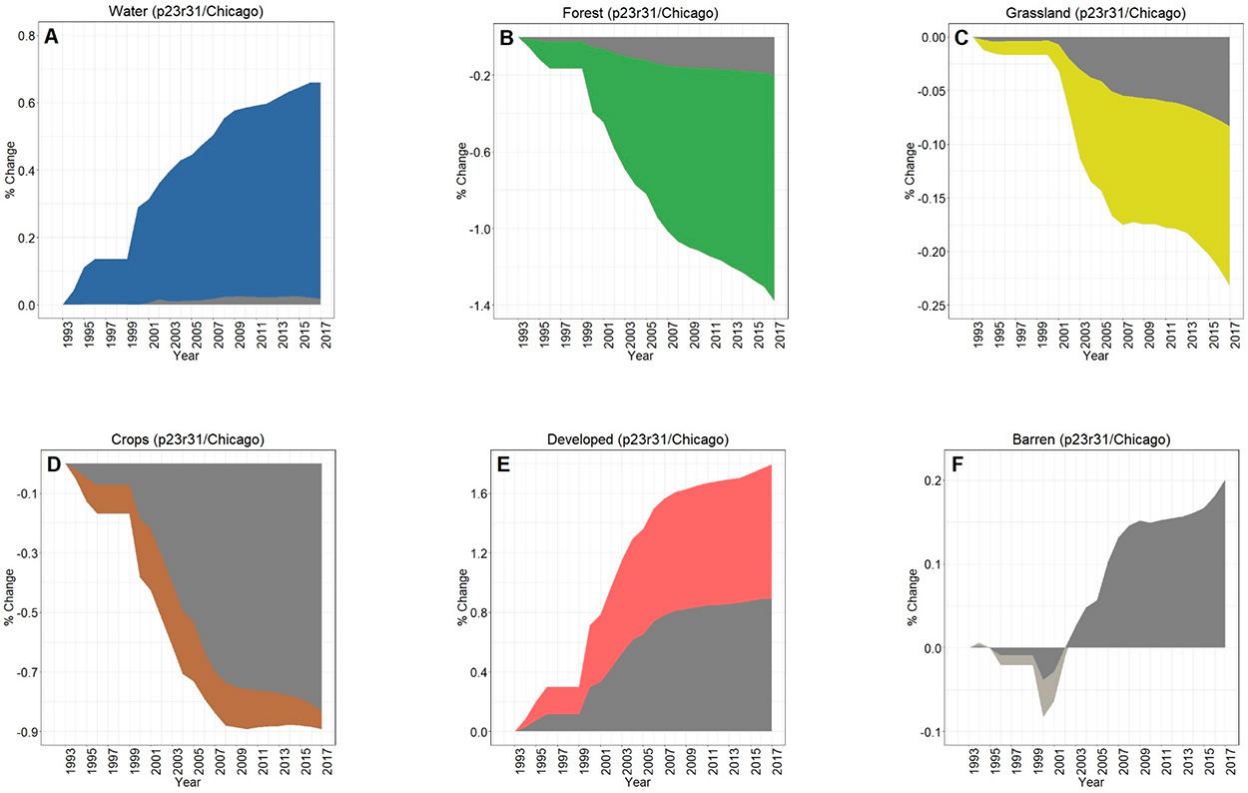
Contrasting LULC change in the areas buffering waters with the Chicago-area footprint-wide change for (**A**) water, (**B**) forest, (**C**) grassland, (**D**) crops, (**E**) developed, and (**F**) barren classes.

**Figure 10.**
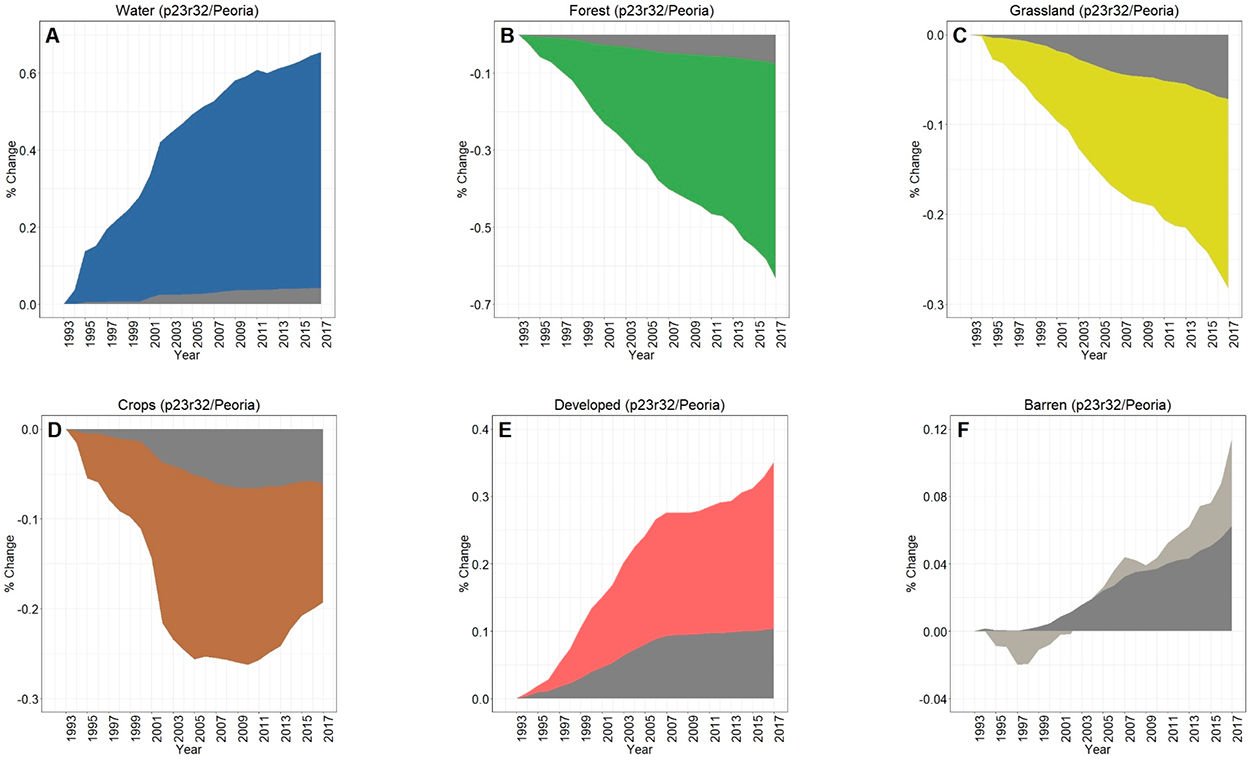
Contrasting LULC change in the areas buffering waters with the Peoria-area footprint-wide change for (**A**) water, (**B**) forest, (**C**) grassland, (**D**) crops, (**E**) developed, and (**F**) barren classes.

**Figure 11.**
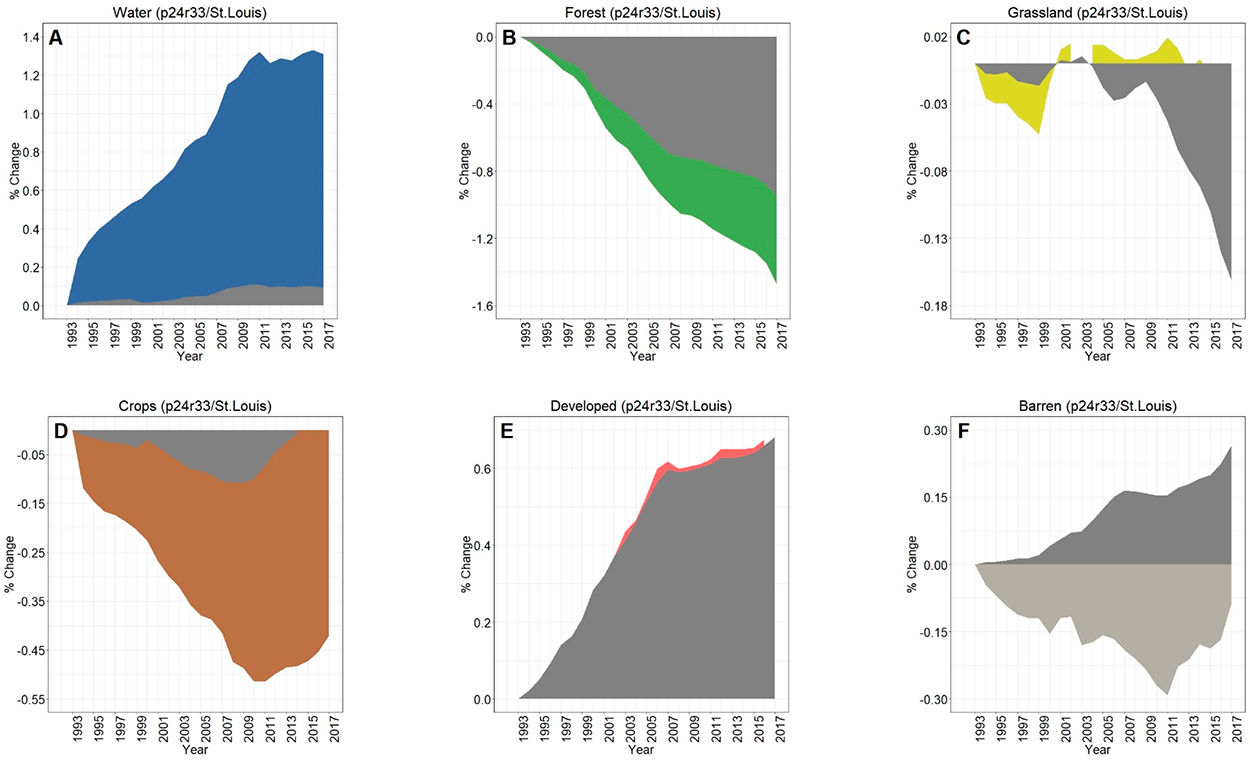
Contrasting LULC change in the areas buffering waters with the St. Louis-area footprint-wide change for (**A**) water, (**B**) forest, (**C**) grassland, (**D**) crops, (**E**) developed, and (**F**) barren classes.

The overall LULC change in buffers surrounding water bodies, given in Figure 8A-C, was typically similar in direction to that occurring in footprint-wide analyses (see Figure 3A-C). However, the magnitude of LULC change in the buffering areas was frequently much greater than the footprint-level analyses across all three footprints, as evidenced when contrasting between LULC change in the footprint and the 90 m surface water buffer for Chicago (Figure 9A-F), Peoria (Figure 10A-F), and St. Louis (Figure 11A-F). For example, substantial water expansion occurred in the Chicago study area buffers at 35 times (35x) the relative footprint-wide rate (0.66% versus 0.02% over 25 years, see Figure 9A). Areas buffering surface waters in the Chicago footprint followed the image-wide trend of increased LULC development over 25 years, yet buffer increases were double the image-wide proportional change (1.8% increase compared to ~0.9% increase, respectively (see Figure 9E)). Over the 25 years of Chicago footprint analyses, forests and grasslands buffering surface waters changed at faster rates (decreasing at ~7x and ~3x, respectively; see Figure 9B-C) relative to the full footprint.

Twenty-five years of Peoria imagery analyses follow a similar pattern, with a nearly 15x increase in surface water into the buffers relative to the footprint-wide analyses (see Figure 10A). Developed lands increased 4x in the Peoria buffers relative to the full footprint-wide change (0.35% versus 0.10%, see Figure 10E), whereas there was a 9x decrease in forested lands (−0.63% versus −0.07%, see Figure 10B) and a 4x decrease in grasslands (−0.28% versus −0.07%, see Figure 10C). This footprint also had an increase in barren lands within the buffers 2x greater than the footprint-wide change (0.12% versus 0.06%, see Figure 10F).

Like Chicago and Peoria, surface waters in the St. Louis footprint buffers increased markedly, 14x greater than the entire footprint (1.31% versus 0.09%, see Figure 11A). St. Louis imagery analyses demonstrated no meaningful differences in developed lands between the buffered and footprint-wide changes over 25 years (0.71% versus 0.68%, respectively, see Figure 11E). Forested lands buffering waters were converted at ~2x the footprint rate (−1.48% versus −0.95% over 25 years, see Figure 11B). Grasslands buffering surface waters were converted more slowly than grassland change across the St. Louis footprint, at 1/5 the LULC change rate (−0.03% in the buffer versus −0.16% at the footprint-level over 25 years, see Figure 11C). Croplands in the buffer and footprint-wide followed a similar decreasing trend through ~2008, increasing from that point such that by 2017 there was a slight increase (0.07%) in footprint-wide cropland abundance (though still a substantial decrease in buffer-area croplands (−0.42%; see Figure 11D)). Barren lands decreased in buffering areas (−0.09%) but switched direction and increased at the footprint-scale (0.27%; see Figure 11F).

Changes in lands buffering surface waters were typically not a one-off event. Of the water buffers that changed LULC during our study period, two changes was the mode within each of the three footprints (e.g., from forest to barren, then from barren to developed); 39.6% of Chicago, 46.2% of Peoria, and 38.6% of St. Louis buffers changed LULC twice during the study period. Three or more cumulative LULC buffering area changes occurred in Chicago (57.5%), Peoria (51.6%), and St. Louis (57.9%) across the 25 year analysis.

#### 3.3.2. LULC Change in Buffers by Water Body Sizes

The vast majority of extant surface waters across all three Landsat footprints were <1.0 ha in size (87% of 51,619 surface waters in Chicago, 85% of 22,186 surface waters in Peoria, 88% of 34,475 surface waters in St. Louis). The smallest size class (< 0.1 ha) was the mode in Chicago (44%) though the second-most abundant size class in Peoria (40%) and St. Louis (42%), after waters between 0.1 to <1.0 ha in both areas (Table 4). The largest waters, those ≥ 100 ha (and excluding Lake Michigan in the Chicago footprint), represented 0.1%–0.2% of the extant surface waters in our study areas.

**Figure 12.**
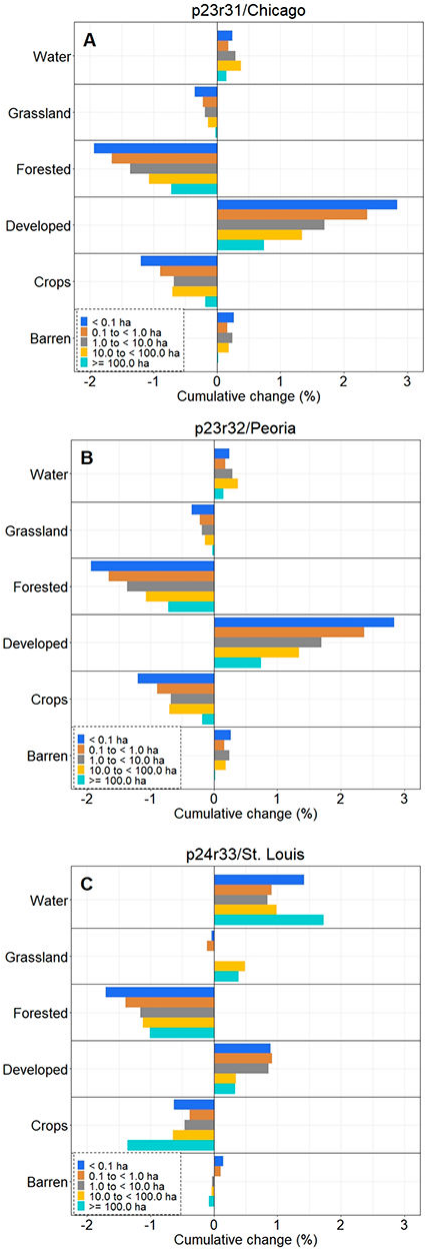
Twenty-five years of LULC change in surface-water buffers binned into five aquatic system size classes across (**A**) Chicago, (**B**) Peoria, and (**C**) St. Louis footprints.

**Figure 13.**
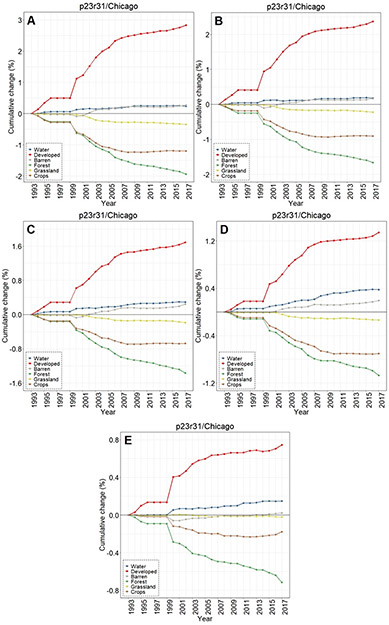
Chicago footprint LULC change over time in surface-water buffers binned into five aquatic system size, (**A**) < 0.1 ha, (**B**) 0.1 to < 1.0 ha, (**C**) 1.0 to < 10.0 ha, (**D**) 10.0 to < 100.0 ha, (**E**) ≥ 100.0 ha. Note the differences in the Y-values between plots.

Buffer LULC change differed substantially depending on the size of the water body being buffered (Figure 12A-C). In the Chicago footprint, developed, croplands, grasslands, and forested lands all had the same approximate stair-step LULC-change trend over 25 y (Figure 12A), with the largest LULC changes in the buffers surrounding the smallest waters and the least change around the largest water bodies. For instance, there was a cumulative 25 y increase in developed lands that was greatest in the buffers surrounding the smallest waters (<0.1 ha, 2.84%) followed by the second-smallest (0.1 to <1.0 ha, 2.37%,), etc. This trend held for decreases in the abundance of forested lands and grasslands; croplands followed suit, but no meaningful difference was discerned between changes in buffers surrounding waters of 1.0 to <10.0 ha (−0.68%) and 10.0 to <100.0 ha (−0.70%). Water (and barren lands) increased over the course of 25 y without any apparent trend associated with size classes in the Chicago footprint. Exploring the temporal nature of the LULC changes, there was a substantive increase in water (in particular), as well as forested and crops around 1999 across all five size classes (Figure 13A-E).

**Figure 14.**
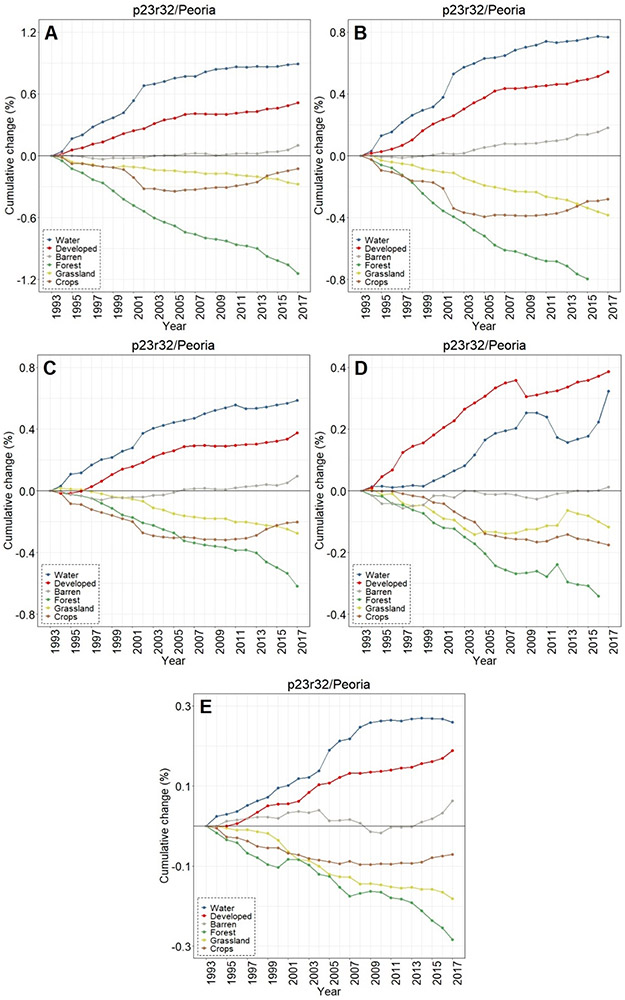
Peoria footprint LULC change over time in surface-water buffers binned into five aquatic system size, (**A**) < 0.1 ha, (**B**) 0.1 to < 1.0 ha, (**C**) 1.0 to < 10.0 ha, (**D**) 10.0 to < 100.0 ha, (**E**) ≥ 100.0 ha. Note the differences in the Y-values between plots.

**Figure 15.**
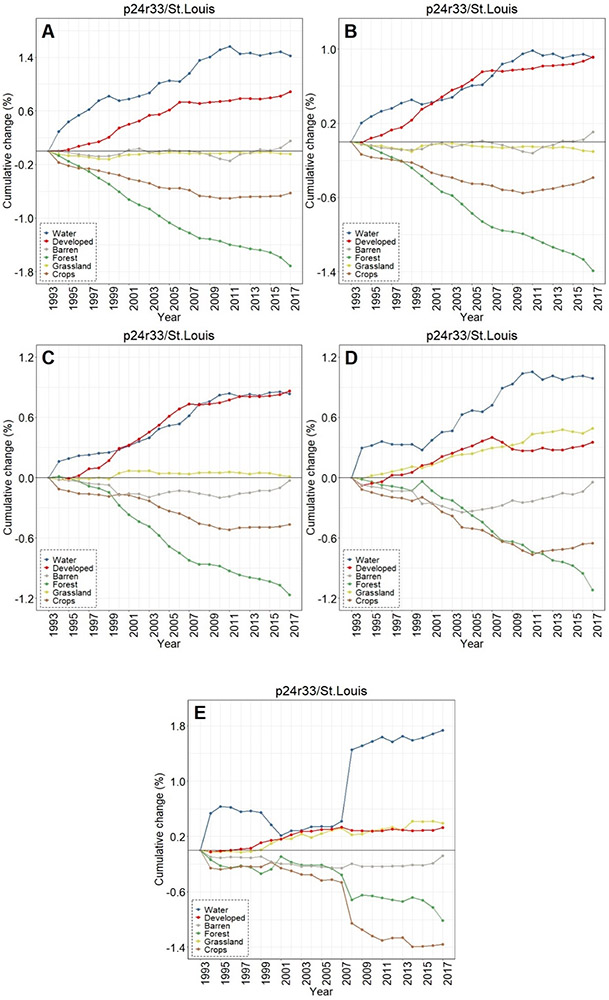
St. Louis footprint LULC change over time in surface-water buffers binned into five aquatic system size, (**A**) < 0.1 ha, (**B**) 0.1 to < 1.0 ha, (**C**) 1.0 to < 10.0 ha, (**D**) 10.0 to < 100.0 ha, (**E**) ≥ 100.0 ha. Note the differences in the Y-values between plots.

The smallest water bodies in Peoria had the largest LULC increase in percentage water, followed in stair-step fashion by the second smallest, etc. (see Figure 12B). The abundance of forested lands decreased in a similar manner, with the greatest change in the areas surrounding the smallest waterbodies and the least change in the largest waterbodies. Developed lands in buffers increased in tandem between waterbodies<1.0 ha (0.52–0.55%) and those between 1.0 ha and <100.0 ha (0.38–0.39%) before halving to 0.18% in the largest waters. Croplands, grasslands, and barren lands did not evince any particular manner of change when contrasted with the binned data. There was a decrease in grasslands in the buffers surrounding the largest waters, starting in 1999, while LULC change in the remaining four size classes demonstrated a more consistent change-trajectory over the 25 y period of record in the Peoria footprint (Figure 14A-E). The largest water bodies (> 100.0 ha) in the St. Louis footprint experienced the greatest increase in water in the buffering areas (1.73%), followed by the smallest water bodies (1.42%); the remaining size classes vacillated between 0.84%–0.99% increases (see Figure 12C). The two largest classes, those water bodies ≥10.0 ha and larger, demonstrated the greatest decrease in croplands in the buffering areas (−0.65% to −1.36%). The smaller water bodies (i.e., the three binned classes <10.0 ha) had the greatest increases in developed lands in the St. Louis footprint (0.86%–0.92%). Forested lands in the St. Louis footprint decreased in a stair-step manner as in Peoria and Chicago, with the greatest decrease in the buffers surrounding the smallest water bodies (<0.1 ha, −1.71%) followed by the next size class (0.1 to <1.0 ha) and so on. CCDC analyses indicated a substantive increase in the abundance of water in areas buffering the largest systems, those ≥ 100 ha, in 2007 (Figure 15A-E).

## 4. Discussion

### Dynamics of LULC Change in the Midwestern US

The progression of LULC change across the Midwestern US has resulted in significant advances in agricultural production, industrialization, and urban growth. Analyzed through the lens of an entire Landsat footprint (or, here, three such footprints), the last 25 years have seen a decrease in forested and agricultural (crop) lands and an increase in developed lands, especially in the urbanizing exurbs of Chicago and St. Louis. This finding follows similar results from LULC change analyses at the conterminous US-scale [62], as well as more regional analyses (e.g., [63,64]). For instance, Maimaitijiang et al. [54] analyzed LULC change in the St. Louis metropolitan area (not a full Landsat footprint) between 1972–2010 at five time-steps (i.e., 1972, 1982, 1990, 2000, and 2010) and reported increases in developed lands and decreases in croplands and grasslands (similar to our study). However, using time-series analyses through CCDC, we found a substantive uptick in agricultural lands around 2010, when their study period ended and near the onset of a near doubling in the per-bushel price of corn [65]. The authors also found decreases in the forested lands and increases in water extent in the greater St. Louis region through 2010 [54], and the change continued apace through to the end of our study in 2017.

Novel to our study of three Landsat footprints covering nearly 100,000 km^2^ is the finding that development is particularly prevalent in the buffered areas surrounding existing small waterbodies—the wetlands, ponds, and lakes providing numerous ecosystem services [19,23,66-68]. Across all three Landsat images, the smallest water bodies (i.e., those < 1.0 ha) experienced the greatest change in the lands buffering them from terrestrial inputs. Furthermore, these changes in the buffers surrounding water bodies occurred at rates substantially faster than the prevailing footprint-wide Landsat changes. Specifically, buffered areas in the Chicago and Peoria footprints converted to developed lands at two to four times the footprint-wide rate, and forested and grasslands decreased between seven to nine times faster (for forested) and three to four times faster (for grasslands) in buffers than occurred across the footprint. Lands buffering water bodies in the St. Louis footprint analysis changed, though at rates similar to the footprint-wide change, with no discernable differences between development (increasing 0.69% in the footprint-wide analysis and 0.72% in the buffers) and relatively minor changes in the forested land conversion in the buffers (~2x the footprint-wide change). Nevertheless, the smaller water bodies in St. Louis (i.e., those <10.0 ha) experienced a higher developed land conversion rate (0.86%-0.92% over 25 years) than the footprint-wide analyses (0.72% over 25 years).

Conversion of the lands buffering water bodies may have profound effects on the waters themselves [29,32,69,70]. For instance, Christensen et al. [71] reported that lands buffering stream networks in agricultural landscapes removed 52% of the edge-of-field nitrogen, a major determinant of aquatic resource eutrophication (e.g., [72]). The buffering areas can filter out pollutants and sediments that affect water quality and aquatic biota, as well as modify temperatures in aquatic systems through shading mechanisms. Supporting the maintenance of aquatic buffers, Wahl et al. [73] found the greatest abundance of pollution-sensitive insect orders Ephemeroptera, Plecoptera, and Trichoptera (“EPT”) in aquatic networks with forested buffers and the lowest in agriculturally cultivated or urban-developed catchments (i.e., those without buffers). Biogeochemical cycling rates within water bodies respond to the differences in loading rates, which can vary depending on the surrounding land uses [70]. Furthermore, the conversion of lands buffering water bodies can increase sedimentation rates and decrease hydroperiods, which can threaten migratory waterfowl and other organisms [26,74-77].

However, while the conversion and development of lands buffering water bodies occurred at elevated rates vis-a-vis the Landsat footprint (in Chicago and Peoria footprints in particular), this study found a substantial expansion of existing waters into the buffering lands across all three footprints analyzed (i.e., buffer expansion rates ranging from 14–35x footprint-wide rates). The smallest waters in Peoria (<0.1 ha) had the greatest expansion, whereas the largest waters in St. Louis (>100 ha) expanded at the highest rates (followed by the smallest waters) in 25 years.

The reasoning behind these surface water expansions remains elusive. Whereas increasing precipitation rates across the Midwestern US [38,78] ascribed to global climate change likely has some effect, the spatio-temporal impacts of precipitation modifications vary across the study areas. For instance, Dai et al. [78] noted increases in Midwestern early growing-season precipitation but cautioned that few trends were statistically significant across their study locations. Furthermore, they found decreases in late growing-season precipitation within Missouri, Illinois, and six other Midwestern states [78]. In a time-series analysis of water body area across the conterminous US, Zou et al. [79] reported waters in the Midwestern US to be increasing in extent and correlated with climate variables (e.g., annual precipitation), though they note that surface-area changes were also associated with dam construction and affected by water withdrawals.

Surface water expansion may also result from the combination of precipitation non-stationarity coupled with anthropogenic alterations to the landscape drainage network. McCauley et al. [32] found that consolidation drainage (wherein many smaller water bodies are connected and drained into fewer larger ones) has increased water surface areas of the Upper Midwestern Prairie Pothole Region (PPR) by 86%; waters in “extensively drained landscapes” were 197% larger than historical records (1937–1969). Similarly, Vanderhoof and Alexander [80] analyzed lake expansion in the Upper Midwestern PPR, reporting substantial lake expansion between 1990–2011. Three large lakes in their study area increased between 237%–578%, subsuming over 7000 surrounding wetlands in the process.

Our analyses determined change is more pervasive in the areas buffering waters across the Midwest than a footprint-wide, regional change analysis would suggest. However, our CCDC analyses were predicated on using the available Landsat archive for the study. These data are remarkable for long-term change analyses, though a caveat is that the analyses were conducted with medium-resolution data (e.g., 30 m pixel size). Our class descriptors incorporated NLCD products using Landsat data, and we binned these data for our purposes. Though our overall accuracy ranged from 81%–89%, certain classes were more poorly discerned than others. For instance, grasslands were often confused (e.g., with developed and/or crops), leading to some uncertainty in the results regarding changes associated with grasslands. While ideally our overall correspondence in our confusion matrices would have been >80% for all classes, our analyses trended within ranges shown for the NLCD products (e.g., NLCD 2001 regional producer’s accuracies for Level I Water of ~60%, or Level I Grassland of ~25%; see, e.g., [81]). The inclusion of ancillary datasets, such as greenness or wetness indices, may improve subsequent classification accuracy in future studies. Furthermore, the increasing availability of high-resolution satellite data and cloud-based computing (Google Earth Engine; see, e.g., [79,82,83]) augurs well for the improved granularity of class and change in the future. For instance, we combined the various developed land cover classes, but future analyses may choose to separate them to more finely discern change or create additional classes to suit emerging needs (e.g., focusing on wetland or forest LULC changes over time, [83]).

Multiple factors affect surface water dynamics, from climate-change induced non-stationarity (e.g., [84]), decadal precipitation cycling, artificial barriers such as roads and berms, and artificial drainage. The influence of these effects, including the magnitude and frequency of storm events as well as the influence of water-retention on surface-water extent and landscape inundation dynamics (e.g., [85,86]), warrant additional investigation.

## 5. Conclusions

The Midwestern US states included in this study have had substantial water body losses since the European settlement. For example, Dahl [39] calculated pre-European settlement wetland losses of −85% for Illinois and −87% for Missouri. However, water body expansion, as evidenced by the increase in water in the buffers across all three Landsat footprints over 25 years, suggests that the prevailing precipitation patterning may be sufficient at the macroscale to maintain a series of inundated water bodies (e.g., for migratory waterfowl). However, this assumption requires the development of local to regional water budgets to more robustly discern the long-term implications of LULC change on inundation patterning and the maintenance of aquatic landscapes (e.g., [67,76]). While the Midwestern US is experiencing increases in surface water extent, our novel analyses have shown that developed lands are encroaching upon existing water bodies. These landscape shifts are very likely modifying the vegetation structure, hydrology, water quality and biotic integrity of the existing water resources. As noted, development or cultivation in areas buffering aquatic resources can result in increased nutrient loading rates [29], changes in hydrology and inundation extent [87], and increased sedimentation [13-15]. The waters analyzed in this study cumulatively perform myriad functions affecting watershed-scale phenomena, such as nutrient retention and processing [88,89]) and river storm-flow attenuation and baseflow maintenance [22,90,91]. Pervasive change to the areas buffering the water bodies within the three study footprints suggests that even when water bodies are not lost to development or cultivation, their functioning (e.g., nutrient processing or hydrologic attenuation) may be modified, thereby affecting down-gradient, watershed-scale rates, storages, and flows of water, solutes, and particulate matter [19,26].

## Figures and Tables

**Table 1.** Chicago footprint confusion matrix contrasting agreement between the three NLCD validation datasets. Overall agreement was consistently ~84%. Note that units are counts of 30 × 30 m pixels, except Producer’s and User’s Accuracy and Overall Agreement, which are percentages.

	LULC	Water	Developed	Forest	Barren	Grassland	Crops
	Water	2015	120	625	24	31	85
	Developed	261	15,191	1056	319	561	2460
	Forest	8	48	4524	8	5	73
	Barren	489	257	5	119	161	457
2001 NLCD	Grassland	135	993	481	66	1370	1814
	Crops	187	671	481	193	540	43,413
	Producer’s Accuracy (%)	65.1	87.9	63.1	16.3	51.3	89.9
	User’s Accuracy (%)	69.5	76.5	76.7	46.1	28.2	95.4
	Overall Agreement (%)	84.1					
	Water	2026	120	610	22	30	81
	Developed	281	15,764	1099	541	601	2699
	Forest	4	59	4454	7	4	58
	Barren	478	241	2	119	154	437
2006 NLCD	Grassland	136	882	456	53	1317	1752
	Crops	185	623	466	105	520	42,870
	Producer’s Accuracy (%)	65.1	89.1	62.8	14.0	50.2	89.5
	User’s Accuracy (%)	70.1	75.1	77.2	48.4	28.7	95.8
	Overall Agreement (%)	84.0					
	Water	2035	111	607	20	35	100
	Developed	307	16,025	1159	618	631	2921
	Forest	10	53	4402	8	4	37
	Barren	480	235	2	108	172	458
2011 NLCD	Grassland	128	778	418	43	1265	1714
	Crops	176	567	453	91	510	42,573
	Producer’s Accuracy (%)	64.9	90.2	62.5	12.2	48.3	89.1
	User’s Accuracy (%)	70.0	74.0	76.5	50.5	29.1	95.9
	Overall Agreement (%)	83.8					

**Table 2.** Peoria footprint confusion matrix contrasting agreement between the three NLCD validation datasets. Overall agreement was consistently ~89%. Note that units are counts of 30 × 30 m pixels, except Producer’s and User’s Accuracy and Overall Agreement, which are percentages.

	LULC	Water	Developed	Forest	Barren	Grassland	Crops
	Water	1068	13	166	3	23	26
	Developed	52	3434	458	120	448	2983
	Forest	2	3	3458	4	0	2
	Barren	381	90	0	26	260	281
2001 NLCD	Grassland	33	206	427	5	1216	839
	Crops	68	748	877	48	744	61,363
	Producer’s Accuracy (%)	66.6	76.4	64.2	12.6	45.2	93.7
	User’s Accuracy (%)	82.2	45.8	77.3	78.8	44.6	96.1
	Overall Agreement (%)	88.3					
	Water	1076	12	145	6	24	36
	Developed	56	3473	364	132	447	3011
	Forest	2	3	3305	6	2	2
	Barren	374	88	0	28	260	277
2006 NLCD	Grassland	33	199	282	8	1209	837
	Crops	61	738	633	39	729	61,122
	Producer’s Accuracy (%)	67.2	77.0	69.9	12.8	45.3	93.6
	User’s Accuracy (%)	82.8	46.4	76.7	75.7	47.1	96.5
	Overall Agreement (%)	88.9					
	Water	1064	11	148	6	28	38
	Developed	60	3486	369	147	454	3041
	Forest	1	2	3289	5	1	2
	Barren	373	83	1	25	258	285
2011 NLCD	Grassland	36	192	285	10	1191	840
	Crops	64	729	629	42	723	61,086
	Producer’s Accuracy (%)	66.6	77.4	69.7	10.6	44.9	93.6
	User’s Accuracy (%)	82.2	46.1	76.6	78.1	46.6	96.5
	Overall Agreement (%)	88.8					

**Table 3.** St. Louis footprint confusion matrix contrasting agreement between the three NLCD validation datasets. Overall agreement was consistently ~81%. Note that units are counts of 30 × 30 m pixels, except Producer’s and User’s Accuracy and Overall Agreement, which are percentages.

	LULC	Water	Developed	Forest	Barren	Grassland	Crops
	Water	2292	57	980	77	57	140
	Developed	55	4622	1585	151	1430	1114
	Forest	10	17	27,031	26	9	11
	Barren	229	208	16	82	1237	760
2001 NLCD	Grassland	40	251	1834	39	9418	1865
	Crops	146	162	896	63	1332	21,011
	Producer’s Accuracy (%)	82.7	86.9	83.6	18.7	69.9	84.4
	User’s Accuracy (%)	63.6	51.6	91.7	56.6	70.0	89.0
	Overall Agreement (%)	81.3					
	Water	2315	60	988	70	52	226
	Developed	62	4779	1594	189	1449	1151
	Forest	13	15	26,820	49	11	10
	Barren	220	235	12	87	1267	731
2006 NLCD	Grassland	45	260	1825	49	9356	1862
	Crops	148	163	883	68	1315	20,874
	Producer’s Accuracy (%)	82.6	86.7	83.5	17.0	69.6	84.0
	User’s Accuracy (%)	62.4	51.8	91.5	58.8	69.8	89.0
	Overall Agreement (%)	81.0					
	Water	2365	50	978	63	53	223
	Developed	69	4884	1628	198	1485	1189
	Forest	13	16	26,712	52	12	9
	Barren	215	217	14	90	1279	724
2011 NLCD	Grassland	54	240	1824	50	9202	1844
	Crops	137	146	880	72	1405	20,861
	Producer’s Accuracy (%)	82.9	88.0	83.4	17.1	68.5	83.9
	User’s Accuracy (%)	63.4	51.7	91.5	58.4	69.6	88.8
	Overall Agreement (%)	80.9					

**Table 4.** LULC change around all study area water bodies was analyzed according to water body size class, ranging from < 0.1 to ≥ 100 ha.

Chicago Footprint				
Size (ha)	Count	Area (ha)	Average (ha)	Standard Deviation (ha)
<0.1	22,877	2058.9	0.1	0
0.1 to <1.0	21,828	7921.3	0.4	0.3
1.0 to <10.0	6019	17,631.6	2.9	2.1
10.0 to <100.0	822	21,582.8	26.3	19.5
≥ 100.0	73	20,612.1	282.4	303.9
All	51,619	69,806.7	1.4	16.1
Peoria Footprint				
Size (ha)	Count	Area (ha)	Average (ha)	Standard Deviation (ha)
<0.1	8821	793.9	0.1	0
0.1 to <1.0	10,120	3785.3	0.4	0.2
1.0 to <10.0	2842	8015.4	2.8	2.0
10.0 to <100.0	363	9247.2	25.5	18.9
≥ 100.0	40	21,826.7	545.7	74.2
All	22,186	43,668.5	2.0	39.0
St. Louis Footprint				
Size (ha)	Count	Area (ha)	Average (ha)	Standard Deviation (ha)
<0.1	14,291	1286.2	0.09	0
0.1 to < 1.0	15,869	5895.4	0.4	0.2
1.0 to <10.0	3920	10,511.2	2.7	1.9
10.0 to <100.0	365	9064.4	24.8	18.4
≥ 100.0	30	6958.1	231.9	209.8
All	34,475	33,715.4	1.0	9.7
